# Case study: The downside of using a worst‐case approach in occupational safety policy as an interpretation of the precautionary principle: Putting the uncertain UXO occupational safety risk into probabilistic perspective

**DOI:** 10.1111/risa.17653

**Published:** 2024-09-17

**Authors:** Marijn Helsloot, Wino Snip, Ira Helsloot

**Affiliations:** ^1^ Faculty of Management Sciences Radboud University Nijmegen Nijmegen the Netherlands; ^2^ TenneT Engineering & Services TenneT TSO B.V. Arnhem the Netherlands; ^3^ Crisislab Renswoude the Netherlands; ^4^ Industrial Engineering Center IMT Albi Albi France; ^5^ Institute of Political Science Technische Universität Darmstadt Darmstadt Germany

**Keywords:** Precautionary principle, Worst‐case approach, Probabilistic Risk Assessment, Unexploded ordnance (UXO), Cable Installation

## Abstract

Unexploded ordnance (UXO) from the World Wars on the North Sea floor pose an uncertain occupational safety risk for dredging and cable installation. At present mitigation strategies are based on an interpretation of the precautionary principle that uses a worst‐case approach, that is, assuming that UXO will be encountered, will explode, and will harm people onboard. We propose a probabilistic framework to estimate the UXO risk. Using this probabilistic framework, we conclude that the UXO risk during cable installation meets the prevailing safety standard in the Netherlands. Furthermore, we demonstrate that the UXO risk is lower than the general maritime risk, that is, the occupational health risk caused by the mitigation is higher than the UXO risk itself. We conclude that even for uncertain occupational risks, such as the UXO risk in the North Sea, a probabilistic analysis can be more instrumental in the decision‐making process on accepting and mitigating risks than using worst‐case scenario thinking.

## 1 INTRODUCTION

UXO from the First or Second World War on the North Sea floor pose an uncertain occupational safety risk for dredging and cable laying. Explosives are still encountered annually during activities in the North Sea. While just a limited number of UXO incidents are known to have happened in the North Sea area over the last decades during fishing, there are, however, no UXO incidents known during dredging and cable laying in the North Sea (Crisislab, 2023).

The present mitigation strategy in the Netherlands for the occupational risk of UXO boils down to having certified companies conducting investigations into the possible presence of UXO on the location because of wartime activities. If there is any possibility of explosives being present that could cause damage when exploding, these certified investigators recommend mitigating the risk. This is in accordance with Dutch occupational health and safety legislation regarding the UXO risk (Working Conditions Decree, article 4.10, 2024). By and large, the current mitigation strategy consists of the following steps: detection of obstacles on the seafloor, identification of these objects, and clearance when they turn out to be UXO.

These explosives left in the North Sea have some “unique” characteristics that make the risk difficult to quantify:

- The locations of dumped explosives are imprecise (such as jettisons), floating mines could have drifted before they did sink, wave interaction during storms could have shifted UXO over the seabed and fishermen may displace explosives with their nets (Expload et al., 2019).
- The explosives dating back to the World Wars have been lying in the North Sea for decades. There are indications that the likelihood of explosion decreases due to aging and seawater exposure (Kroon et al., 2015).
- The impact of an underwater explosion on ongoing operations remains unknown due to the absence of incidents. The available scientific studies (DNV GL Maritime, 2021; Szturomski, 2015; Van Aanhold et al., , 2017) only assess the possibility (yes or no) of sinking or injury, adhering to the worst‐case approach. The probability of injury or fatality remains uncertain.

Consequently, the probability of an explosion with damage following the encountering of an explosive has often been classified as “not excluded.” This leads to a standard recommendation for further investigation and detection of any location.

The practice of taking such precautions based on a worst‐case scenario when faced with uncertain risks is an interpretation of the well‐known precautionary principle. The principle states that in the case of risks with potentially catastrophic and irreversible effects, preventive measures should be taken to mitigate the risk (Aven, 2023; Vanem, 2012). There are many diverse interpretations of the precautionary principle (Aven, 2023; Hanenkamp, 2015). Broadly speaking, weaker forms (such as simple preventive strategies like a safety margin) or stronger forms (such as requiring the implementation of best‐available‐technique measures or banning activities in the presence of uncertain risks) of the precautionary principle can be distinguished.

The broad application of the precautionary principle has faced criticism (Aven, 2023; Forrester & Hanekamp, 2006; Sunstein, 2002). An incorrect application of the precautionary principle ultimately could lead to more *statistical deaths*. Sunstein (2002) used the concept of statistical deaths to compare risks; it involves assessing the overall health impact rather than focusing on individual fatalities. Mitigation measures also bring new dangers or maintain existing risks. Furthermore, since resources can only be utilized once, other existing risks remain because the resources cannot be used elsewhere.

Originally, the principle was not intended to eliminate every possible risk. The principle should only by applied in situations where “the risk threat is considered high” and “the uncertainties are scientific” (Aven, 2023). In that case, there should also be at least a scientific discussion about the probabilities, uncertainties and consequences (Pieterman & Hanenkamp, 2002). In the Netherlands, however, we see that the interpretation of this principle has led to the application of the “precautionary worst‐case approach” for occupational health and safety policy: in the face of uncertain risks, the responsible party must either minimize risks by implementing best‐available techniques or refrain from the activity altogether (see Section 2).

There are, however, other approaches to addressing occupational safety risks, such as the *absolute risk criteria* (where the risk is evaluated against a predefined standard) and the *As Low As Reasonably Practicable (ALARP)* principle (which also considers both costs and benefits) (Aven & Vinnem, 2005; Vanem, 2012). These approaches are characterized by being based on scientific risk analysis.

A classic example in the occupation health domain is method developed by Fine and Kinney (1971), where values are assigned to *consequences*, *exposure*, and *probability*, and then multiplied together to determine the level of risk. One or more experts determine the values of these risk components. A more or less equivalent approach involves multiple experts providing a quantitative assessment of the risk. In the United States, this method for conducting a risk analysis has been applied to UXO risk, where multiple experts provide an assessment of the risk (Gibson et al., 2008). For both approaches, however, there is no consensus on the likelihood of UXO explosion (MacDonald et al., 2008). Furthermore, experts easily overestimate factual probabilities (Helsloot & Vis, 2020; Skjong & Wentworth, 2001).

An alternative approach to dealing with uncertainties in risk analysis is using statistics (e.g., Bayesian method) or historical data (Aven, 2016; Bowers, 1994; Sánchez et al., 2017). Following Kaplan and Garrick (1981) this is about examining both the actual probability of occurrence and the actual probability of fatality. The use of statistics in risk analysis can be contentious when there are significant or profound uncertainties in risks that are not easily justifiable, or when there is a lack of data (Aven, 2016).

This article explores whether a more well‐founded and probabilistic risk analysis is also possible for uncertain occupational safety risks, as an alternative to the “precautionary worst‐case approach.” We apply this alternative approach to the case of installing cables in the North Sea. Cables are buried into the seabed to protect the cables against external threats. A cable burial tool as for instance a jet trencher is used to bury the cable into the seabed. Prior to the cable installation activities, surveys are conducted to detect obstacles that could hamper installation. Obstacles which are considered to be a potential UXO must be identified and cleared.

A well‐founded risk analysis is especially essential in this situation because working in the North Sea already carries a high occupational risk (e.g., IMCA, 2021) so minimizing exposure has serious advantages. Moreover, significant societal resources (money, personnel, material and time) are invested in these mitigation measures. Detecting, identifying and clearing UXOs incurs costs in the tens of millions of euros per project. Using this broader perspective, it is crucial to assess the UXO related risk within the broader context of other occupational risks.

## 2 SAFETY STANDARD IN THE NETHERLANDS

From a public administration point of view, the governmental risk policies show an interesting dichotomy. Since 1989, the formal governmental policy has used a norm for acceptable risks of 10^–5^ per year (1 in 100,000 years) for risk categories (Ministry of Housing, Spatial Planning and the Environment, 1989). This safety standard is also applicable to working with hazardous, carcinogenic substances (Health Council of the Netherlands, 2021).

However, for not so easily quantifiable risks governmental actors like the Netherlands Labour Inspectorate fall back to a zero‐risk policy that gives rise to a worst‐case approach such as the case of UXO at hand. The labour legislation in the Netherlands states that occupational safety risks must be made transparent in a *Risk Inventory and Evaluation* (RI&E), and measures must be taken to mitigate these risks (Working conditions Act, Article 5, 2023). No safety standard is established for these safety risks. This results in a zero‐risk approach and therefore precautionary worst‐case approach as an interpretation of the labour legislation. For example, the legislation on UXO states (Working Conditions Decree, Article 4.10, 2024):

If further investigation reveals that there is a risk to the safety or health of employees due to the presence of unexploded ordnance, those unexploded ordnance will be located or other appropriate measures will be taken to prevent this hazard.

Another example is the use of quartz‐containing ballast in railway works. Quartz dust can pose health risks with prolonged exposure, resulting in the establishment of threshold limits (Dutch Expert Committee for Occupational Standards, 1992). Some workers were insufficiently protected and were exposed to excessively high concentrations. The Labour Inspectorate states therefore that a switch must be made to much more expensive quartz‐free ballast, noting: “We do not consider the price of that; as far as we are concerned, health has no price” (NOS Nieuws, 2022).

Although there is overlap between the domains of environmental safety and occupational safety, the safety standard of 10^–5^ per year (1 in 100,000 years) is not officially established for occupational safety issues. In the absence of a better‐fitting standard, we therefore use the 10^–5^ per year safety standard as a threshold value in this study.

As a side note, in the international maritime sector, a lower standard is generally used: the probability of an individual's fatality being less than 10^–4^ (1 in 10,000 years) for the crew is deemed acceptable (Aven & Vinnem, 2005; Paté‐Cornell, 2002; Vanem, 2012).

Not a norm as such, but the product of logical thinking is that the risk should not be greater than the risk introduced by the mitigation measures. This aligns with the perspective of Aven and Vinnem (2005) that the focus should not be on an absolute risk standard but on the cost‐effectiveness (in a wide sense) of measures. In this case, implementing mitigation measures requires additional efforts in the North Sea. If the UXO risk is lower than the risk of fatality from regular activities or the general maritime risk in the North Sea, implementing mitigation measures would be counterproductive.

In the Netherlands, an average of approximately three employees per year die on a ship, excluding fishermen (Dutch safety board, 2013–2020). There are approximately 60,000 people working directly in this sector (Van den Bossche et al., 2023). We included the categories maritime shipping, inland shipping, offshore and construction on water. Therefore, the individual risk of working at sea is approximately (3 / 60,000 =) 5 × 10^–5^ per year (1 in 20,000 years). International figures from IMCA (2021) and ESMA (2019) show similar orders of magnitude for the risk: 3 × 10^–5^ to 5 × 10^–5^ per year. Mitigating this risk is therefore “reasonable” when the UXO risk is higher than 5 × 10^–5^ per year (1 in 20,000 years).

## 3 RISK ASSESSMENT METHOD FOR UXO

This study explores whether it is possible to quantify the UXO risk in the North Sea and compare it to the safety standard in the Netherlands. In this exploratory study, the probability of damage or injury is explicitly not considered.

The following formula has been used to quantify the UXO risk:

$$p_{\mathrm{fat}|\mathrm{UXO}}=p_{\mathrm{enc}}\times p_{\mathrm{expl}|\mathrm{enc}}\times p_{\mathrm{fat}|\mathrm{expl}}$$

*p*_fat | UXO_ = Probability of a fatality as the result of an UXO related incident
*p*_enc_ = Probability of encountering a UXO at sea
*p*_expl |enc_ = Probability of an explosion of the UXO as result of the encountering
*p*_fat | expl_ = Probability on a fatality as the result of an explosion of the UXO

This formula is simple yet meaningful. In simple terms, if any of the three probabilities is sufficiently small, there is no need to further consider the other probabilities.

To address uncertainties, confidence intervals or probability scales are often recommended and can support policy decisions (Paté‐Cornell, 1996; Van Erp & Van Gelder, 2008). Therefore, we calculate a confidence interval regarding the probability of explosion (see Section 4.2). However, using the boundaries of confidence intervals for decision making has faced criticism (Morey et al., 2015). The use of confidence intervals can be problematic when there is a lack of data or no incidents of failure, resulting in a wide interval that may lead to a worst‐case approach. We suggest using the mean instead of (the upper bound of) confidence intervals. This is in accordance with for example the Dutch law for calculation of the effects of earthquakes caused by gas production (Article 1.3a.2, lid 3 of the Mining Regulation, 2024).

In this study, we thus do not use technical models but rely on statistical methods. The datasets used are the raw dataset from the Royal Netherlands Navy, containing over 1,500 registrations of UXO after 2005 (Helsloot & Helsloot, 2023; Authorised summery in supplementary file), the public data set from dredging activities in the North Sea (ICES, 2016, 2019) and dataset with incidents reported in the Dutch media involving UXO (Crisislab, 2023). Our assumption is that UXO explosions are rare and therefore reported in the media.

## 4 RESULTS OF THE CASE STUDY

This section provides a brief overview of how the three component probabilities were calculated. For a more detailed description of these probabilities, the research report contains a comprehensive explanation (see: Helsloot & Helsloot, 2023).

### 4.1 Probability of encountering

Prior to conducting activities in the North Sea, surveys are carried out. If an explosive is found during the survey, it is reported to the Royal Netherlands Navy. After the surveys, 110 objects were reported, of which 52 were eventually located and confirmed to be UXOs.

Based on the dataset from the Royal Netherlands Navy, it was possible to identify 11 different survey projects. To calculate the density of explosives per km^2^, the coordinates of the explosives were connected in chronological order and multiplied by the surface area that a ship can investigate. In total, at least 55 km^2^ was surveyed. This approach results in an average density of UXO for the Dutch North Sea.

The density of explosives, and consequently the probability of encountering them, is approximately 0.9 per km^2^ (Table 1). In other words, when 1 km^2^ of seabed is disturbed, an average of 0.9 UXO will be encountered.

**TABLE 1:** Encountered objects in ‘clustered’ surveys and the number of UXO per km^2^.

UXO	Number of UXO per km^2^
Mine	0.638
Aerial bomb	0.207
Depth charge/torpedo^*^	0.020
Projectile	0.061
Total	0.926

* We combined torpedoes and depth charges because these objects are mostly encountered in the same area, former convoy routes.

### 4.2 Probability of explosion

The probability of explosion was determined in two phases. First, the activities that occurred before the implementation of the identification and clearance of explosives were examined. These mitigation measures were introduced around 2015. From 1974 to 2014, approximately 897.9^*^ 10^6^ m^3^ of sand were dredged in the Dutch North Sea (ICES, 2016, 2019). Dredgers require about 1.62 km^2^ of work area to obtain 1^*^ 10^6^ m^3^ of sand. Thus, a total of 1,455 km^2^ of North Sea surface area was dredged. Based on the density (see Section 4.1), it was estimated that approximately 1,350 UXOs were encountered during dredging. However, no explosions have been reported, indicating that the probability of an explosion is at least less than 1 in 1,350.

Based on the expected number of encountered UXOs, we can calculate the 95% confidence interval (CI) or probability scale. In cases where the probability of failure is small (as in this study: *r* = 0 in *n* trials), the probability scale can be calculated according to: (Van Erp & Van Gelder, 2008)

$$95\%\;CI=\frac{1}{n+1}\pm 2\sqrt{\frac{1}{n\left(n+1\right)}}$$

Second, the Netherlands Organization for Applied Scientific Research (Kroon & Bouma, 2020) conducted research on the probability of an explosive unexpectedly detonating due to activities in the North Sea. This concerns explosives that are still in pristine war‐condition. According to this study, the probability of an explosive detonating through jet trenching is 2.5 to 8.2 times lower (depending on the explosive) than through hopper dredging. It is reasonable to assume that the probability of explosion when installing cables is about 2.5 to 4.0 times lower (excluding projectiles) than when dredging (see Table 2).

**TABLE 2:** Probability of explosion for jet trenching.

UXO	P(explosion) for dredging per encountered UXO	95% Confidence interval for dredging^*^	Difference in trigger likelihood (factor)	P(explosion) for trenching per encountered UXO
Mine	<1.1 × 10^–3^	[0.000, 0.003]	4.0	<2.7 × 10^–4^
Aerial bomb	<3.3 × 10^–3^	[0.000, 0.010]	2.9	<1.1 × 10^–3^
Depth charge/torpedo	<3.4 × 10^–2^	[0.000, 0.101]	2.5	<1.4 × 10^–2^
Projectile	<1.1 × 10^–2^	[0.000, 0.034]	8.2	<1.4 × 10^–3^

* Based on the formula above, where *n* is the number of expected encountered UXO calculated by density (see Section 4.1) times 1,455 km^2^ (e.g., the *n* for Mine is calculated as 0.638 ^*^ 1,455 = 928).

### 4.3 Probability of fatality

Several studies have been conducted to assess the effect of an underwater explosion (DNV GL Maritime, 2021; Szturomski, 2015; Van Aanhold et al., 2017). For cable burial tools as jet trenchers, not only the water depth but also the distance between the trencher and the ship must be taken into account. Based on these studies, it can be concluded that, with a water depth of 20 m, only aerial bombs of 1000 lb or more, and mines and depth charges with a TNT mass of 250 kg or more pose a risk of injury to cable installers. For smaller UXO the buffer of water between the UXO and the ship eliminates the risk on an incident resulting in a fatality.

The dataset from the Royal Netherlands Navy shows that only 27% of aerial bombs weigh 1,000 lb or more. Projectiles, contact mines and depth charges generally have less explosive mass than 250 kg TNT. Torpedoes and influence mines often have a larger explosive mass than 250 kg TNT (based on the overview of encountered bombs in the German Bight, received by internal communication). The dataset from the Royal Netherlands Navy indicates that 33% of the encountered mines are influence mines, and 40% of the encountered depth charges/torpedoes are torpedoes (Table 3).

**TABLE 3:** Probability of fatality after explosion UXO.

UXO	*P*(incident)	*P*(fatality | incident)	*P*(fatality)
Mine	0.33	0.117	0.039
Aerial bomb	0.27	0.117	0.032
Depth charge/torpedo	0.40	0.117	0.047
Projectile	0	0.117	0

The studies have focused on the probability of injury rather than the probability of fatality. Based on the incidents reported by the Dutch Safety Board (2013‐2020), 11.7% of occupational health incidents in the maritime sector result in fatalities. Due to the absence of other data, we assume the probability of fatality following an explosion is the same as the probability of fatality following injury from accidents in regular maritime incidents.

### 4.4 Probability of a fatality as the result of an UXO related incident

The risk per square kilometre is calculated by multiplying the three probabilities (Table 4). The risk is then at least less than 2.72 × 10^–5^ per km^2^.

**TABLE 4:** Risk assessment per km^2^.

UXO	P(encounter per km^2^)	P(explosion) for trenching per encountered UXO	*P*(fatality)	Individual risk per km^2^
Mine	0.638	<2.7 × 10^–4^	0.039	<6.71 × 10^–6^
Aerial bomb	0.207	<1.1 × 10^–3^	0.032	<7.31 × 10^–6^
Depth charge/torpedo	0.020	<1.4 × 10^–2^	0.047	<1.32 × 10^–5^
Projectile	0.061	<1.4 × 10^–3^	0	0
Total	–	–	–	<2.72 × 10^–5^

Now the risk has been calculated per km^2^, also the individual risk for employees on board a ship can be calculated. In this case study, we assume:

- a water depth of at least 20 m,
- the electrical cables, which are at most 30 cm wide and cause an average disturbance of 60 cm of seabed, are buried at a speed of approx. 200 m/h using a jet trencher (data received from Dutch offshore grid operator), and
- 24‐hour “on‐and‐off” shifts, with a maximum of 173 working days per year (State Supervision of Mines, 2014).

So, an individual employee disturbs (173 days, 24 h, 0.00012 km^2^ per hour) 0.498 seabed per year. Note that a person may not work more than 12 h per day. Therefore, we consider the presence on a ship as a risk rather than working on a ship. The individual risk is then at least less than 1.35^*^ 10^‐5^ per year.

## 5 CONCLUSION

The individual risk for employees to die from UXO related risks during cable installation is less than 1.35 × 10^‐5^ per year (< 1 in 75,000 years). Given the conservative assumptions used in the calculations we conclude that this risk may be considered to meet the safety standard in the Netherlands of 1 × 10^–5^ per year.

Furthermore, the UXO risk during cable installation in the North Sea is lower than the general maritime risk, which is estimated at 5.0 × 10^‐5^ per year (1 in 20,000 years). By implementing mitigating measures, seamen are thus exposed to a greater risk than the risk being mitigated poses to their colleagues.

Even if we use the upper bound of the 95% confidence interval as the probability of explosion (adjusted by the factor representing the difference in trigger likelihood presented in Section 4.2), the risk remains less than the general risk of working at sea. In this case, the individual risk is less than 4.02 × 10^−5^ per year (< 1 in 25,000 years).

## 6 DISCUSSION

The current practice in the Netherlands is to prevent uncertain occupational risks as much as possible with new technical measures. One of the uncertain risks that is not accepted in the Netherlands is the potential presence of UXO from the World Wars in the North Sea. To mitigate this uncertain risk, societal resources, such as money, personnel, and time, are used to detect, identify, and clear explosives.

This study has explored the possibility of dealing probabilistic with uncertain occupational risks as an alternative to the “precautionary worst‐case approach.” This alternative approach could initiate the discussion about scientific uncertainties regarding the specific risk. A scientific discussion should ideally be a key characteristic of the precautionary principle (Aven, 2023; Pieterman & Hanenkamp, 2002). Moreover, this study thus also reflects on the precautionary measures taken in the past.

### 6.1 Limitations

We see two types of limitations in this approach. First, the study has taken a broad look at the risks of UXO in the North Sea. In reality the density of UXO in the North Sea however cannot be considered equal everywhere. The density, and therefore the probability of encountering UXO, may vary from one area to another in the North Sea. There is a relation between the location of former minefields at sea and the density of non‐cleared sea mines in and on the seabed, as example (Möller, 2023). Furthermore, we assumed a water depth of at least 20 m, which is practically the case for most locations in the North Sea, except for nearshore operations. Therefore, the study cannot be directly projected onto a project level. A more precise understanding of the probability of the distribution of UXO in the North Sea could provide a solution. Moreover, please note that in this study, we actually examined the density of UXOs and equalled that to the probability of encountering them. A more precise probability of encountering one or more UXOs could be calculated using the Poisson distribution.

Second, we determined the probability of an explosion after encountering UXO based on the number of encountered explosives and the reported incidents in the media. Since no incidents with UXO have been reported during cable installation, we could only calculate a “less than” probability. We therefore tend to overestimate the actual risk. Moreover, in line with the criticisms of Morey et al. (2015), the confidence interval provides a further overestimation of the risk. A tentative comparison with fishing, where some incidents have been reported and hundreds of explosives are encountered each year, may provide insight. The probability of an explosion can be estimated at approximately 1 × 10^–4^ per explosive (Helsloot & Helsloot, 2023). The incidents involving UXOs with Dutch fishermen indicate that the fatality risk materializes only when the explosive is brought on board.

### 6.2 Implications

Even with uncertain risks, a quantitative risk analysis can help in decision‐making process (Apostolakis, 2004; Sunstein, 2002). Based on this statistical method, the quantified risk was determined, and it “appears” to meet the prevailing safety standard in the Netherlands. The phrase “it appears to meet” is used deliberately because there is no quantitative standard for occupational safety accidents in the Netherlands. The standard that is used in this study comes from environmental safety and is already being used for hazardous substances. As long as a zero‐risk approach remains the starting point, quantifying risks alone is meaningless. The collision between these realities is referred to by Slovic et a. (2004) as “risk as politics.” This aligns with the notion that risks have both factual and valuational components (Hansson, 2010; Pate‐Cornell, 2002). Therefore, more than just quantifying a risk is needed for its acceptance. What constitutes a “not unacceptable risk” will need to be determined.

In this research, only occupational safety risks were considered, which means that other elements, such as the costs of mitigation measures, but also potential litigation and compensation after an accident, were not taken into account. Detecting, identifying, and clearing UXOs incur costs in the tens of millions of euros per project. Sunstein (2002) and Kaplan and Garrick (1981) conclude that “quantitative safety” must be considered in its context. The ALARP principle may offer a solution (Vanem, 2012). Now that we know the probability of fatality or the individual risk posed by UXOs, we can also assess what a “reasonable” investment is to manage this risk. What is reasonably must then be determined and is a political issue. In this study, we did not take into account the number of individuals exposed to the risk. In theory, it could be that a small group is exposed to a risk so that a larger group can work safer. In practice, it appears that the number of individuals on an Identification and Clearance (ID&C)‐ship is fewer than on a cable installation ship, but more days at sea are required to mitigate the risk than to install the cable.

Additionally, this study did not examine what a reasonable investment would be to mitigate the risk. Sunstein (2002) proposes a democratically chosen Value of Statistical Life. The Value of Statistical Life is estimated at 6.7 million for an employee (Viscusi & Aldy, 2003). In the Dutch healthcare context, a maximum amount of €80,000 per gained healthy life year has been proposed (Council of Public Health & Society, 2006). With these mentioned “reasonable amounts for safety”, the current costs, tens of millions of euros per project, seem unjustifiable for mitigating the small individual risk.

We conclude that even with uncertain occupational risks, such as the UXO risk in the North Sea, a probabilistic analysis can assist in the decision‐making process and considerations for mitigating risks. The use of best‐available‐technique measures to manage uncertain risks also has a downside. This study illustrates this effectively: when mitigating the UXO risk during cable installation in the North Sea, the occupational safety risk is higher than the UXO risk itself.

## CONFLICT OF INTEREST STATEMENT

The authors declare no conflicts of interest.

## Supporting information



Supplementary file
Link to authorized summery of dataset with encountered explosives from Royal Netherlands Navy.

## Data Availability

The raw data was obtained through the Royal Netherlands Navy. In the supplementary file, you will find an authorized summary of the dataset. The other data sources are available online and have been referenced in the article.
