# Supplementary material for: Case study: The downside of using a worst‐case approach in occupational safety policy as an interpretation of the precautionary principle: Putting the uncertain UXO occupational safety risk into probabilistic perspective
Source: Risk Anal. 2024 Sep 17;45(5):973–80. doi: 10.1111/risa.17653 (PMC12087751; doi:10.1111/risa.17653)
Supplement: Supplementary file 1 — Supplementary file Link to authorized summery of dataset with encountered explosives from Royal Netherlands Navy. [file RISA-45-973-s001.pdf]

The core data brought together

Encountered UXO

Registrations

To make the risk assessment, the dataset of the Royal Netherlands Navy has been the primarily basis.

Royal Netherlands Navy dataset

The Royal Netherlands Navy (RNN) dataset is provided to RWS by the NATO Mine Warfare School EGUERMIN, and contains the explosives that have been cleared by the Dutch and Belgian navy within the framework of Operation Beneficial Cooperation. This operation encompasses the clearance of mines that have been encountered by civilian vessels and during military exercises.

Civilian vessels that encounter mines or other explosives (should) report this to the Coast Guard, after which a RNN minehunter is deployed to clear the explosive. Details of encountering these mines are logged from report to clearance by the Coast Guard, forming the RNN dataset. This dataset is the only comprehensive dataset containing UXO encountered in the Dutch North Sea.

Whilst comprehensive, the dataset has several drawbacks:

- Part of the report to the coast guard is an identification of the encountered UXO by the reporting party. A silhouette chart showing the most common types of UXO aids civilian parties in the identification. After the report the coast guard often advises the vessel crew to throw the UXO overboard with a sonar reflector for easy retrieval by the RNN. Clearance by minehunters often takes place with divers or ROVs without detailed examination and identification. Since fishermen are not EOD specialists, the identification may be lacking in detail or false. While it is hard to confuse a contact mine with a torpedo, ground mines and aerial bombs may be hard to tell apart, causing an uncertainty in this dataset.
- The mentioned location of encounter is not the location where the UXO was originally situated. Fishermen only encounter UXO after reeling in the nets after dozens of kilometres of trawling. Dredgers encounter UXO after bringing in the dredge head, which may be after kilometres of dredging the seabed.
- Many reported objects are lost after reporting whilst not cleared by the dispatched navy vessel. This may be caused by burial, adverse weather conditions or the absence or loss of the sonar reflector in the process.

To make the database more accessible for Crisislab, RWS edited the raw data provided by EGUERMIN. The reports were categorized in five categories (fishing, dredging, survey, military and other) based on the reporting type of vessel. The reported UXO are categorized based on the reported silhouette, eliminating detail-level mistakes in identification (i.e., all aerial bomb silhouette reports are grouped as 'aerial bombs'). Finally, the reported coordinates were converted to a GIS-compatible format, enabling geographic analysis of the dataset.

Royal Netherlands Navy's dataset contains the following registrations:

UXO	Registrations
Mine (LMB)	19
Mine (contact)	147
Mine (ground)	48
Unknown	174
Aerial bomb	709
Mine (type unknown)	16
Depth charges	50
Projectiles	273
Other	41
Torpedo	34
Mine clearing charge	6
Scrap	8
**Total**	**1,525**

Table 1: The registrations of the encountered UXO.[1]

In total 94% of the registrations were made after 1-1-2005. The majority of the registrations (65%) were made by fishermen.

Activity[2]	Before 1-1-2005	After 1-1-2005	Year unknown	All
Fishing	82	906	5	993
Dredging	3	389	0	392
Military	2	8	1	11
Survey	0	119	0	119
Other	1	9	0	10
**Total**	**88**	**1,431**	**6**	**1,525**

Table 2: Registrations per activity.

An important reason to start systematically registering UXO appears to be the incident with the fishing vessel 'Maarten Jacob' on 6 April 2005. From 1 January to 6 April 2005, only 4 UXO were registered, while 237 UXO were registered in the period from 7 April 2005 to 31 December 2005. The increased registration of UXO encountered during dredging (from 2008) and during surveys (from 2014) also seems to contribute to the number of UXO registrations.

[1] Be aware that these registrations are done by the Royal Netherlands Navy. Beached UXO are not included in dataset and are not part of this research.

[2] The distinction is based on the name of the vessel that reported the UXO.

Encountered UXO - fishery

The 'explosion' of registrations (by fishermen) after April 2005 indicates that not all encountered UXO will always be registered. We suppose that the two months after the incident in 2005 (the period April-May) are representative for 'encountered UXO by fishermen'.

Jan	Feb	Mar	Apr	May	Jun	Jul	Aug	Sept	Oct	Nov	Dec
0	1	2	61	52	29	16	19	18	18	15	8

Table 3: Registrations of UXO by fishermen in 2005.

This means that, in a 'normal year', there will be 678 UXO encountered by fishermen:
*Formula: (61 registrations April 2005 + 52 registrations May 2005) / 2 months * 12 months = 678 UXO.*

The fishing fleet in 2005[3] was:

- 15 large scale fisheries and 355 cutter fisheries = 370 vessels.
- 165 shrimp fisheries (estimation, based on Quirijns et al., 2021).
- 0 pulse fisheries.

So: 370 vessels (all) – 165 vessels (shrimp) – 0 vessels (pulse) = 205 vessels that can encounter UXO.

With other words: fishermen encounter (at least[4]) 3 or 4 UXO per year on average.

Furthermore, it must also be noted that it may be possible that some fishermen encounter fewer UXO (after 2006) as a result of a new method of fishing. Quite a few vessels were equipped with a so-called electric pulse. Electric pulse fishing has been used from 2006 till 2021. While pulse fishing only electric pulse conductor wires are dragged over the seabed with some rigging to support the fishing nets and to keep the nets open. This method does not scrape objects from the seabed, as it relies on fish to jump up from the seabed, as a result of the electric pulses, and thus ending up in the nets.

Categorisation

We differentiate the following objects for the risk assessment:

- Mines.
- Aerial bombs.
- Depth charges and torpedoes.[5]
- Projectiles.

The objects 'scrap', 'other', and 'mine clearing charges'[6] are not included in this research. Moreover, we removed the empty explosives and 'wrong registrations' (e.g., Non-Mine, Mine-Like Bottom Object (NOMBO)) that sometimes has been registered as UXO.

---

[3] Agrimatie (n.d.).*Visserij in cijfers*. Retrieved November 22, 2021, from https://agrimatie.nl/. For shrimp fisheries: we compared the data from 2005 with the data (p. 17) in the report of Quirijns et al. (2021). *Beschrijving garnalenvisserij: Huidige situatie, knelpunten en kansen*.

[4] In the report after the latest UXO-incident in the North Sea (incident of the OD-1 Maarten Jacob), it is posed that a fisherman catches one or two bombs *per week* at some locations. In an interview held for this report a fisherman told us that he encounters bombs on regular basis, i.e. a few a year.

[5] We combine depth charges and torpedoes because these objects are encountered more or less equally often and in the same area.

[6] We removed mine clearing charges since these explosives are rarely encountered.

Proportions

In this study we only include 'real' UXO (so we excluded scrap, other, wrong registrations, etc.). Only (1,275 / 1,525 =) 84% of the registered objects is a *real* and identified UXO. At least 5% of the encountered objects is not an explosive.

UXO	Fishing	Dredging	Survey	All
Unknown	46	83	31	167
Wrong registration, scrap, etc.	34	14	33	83
Identified UXO	913	295	55	1,275
**All registrations**	**993**	**392**	**119**	**1,525**

Table 4: Encountered UXO (unknown, wrong registration or UXO).

A distinction can be made between the activities of fishing, dredging, and surveying.[7]

UXO	Fishing	%	Dredging	%	Survey	%	All activities	%
Mine[8]	165	17%	8	3%	40	45%	217	16%
Aerial bomb	660	70%	29	9%	11	13%	706	52%
Depth charges/ torpedoes	72	8%	9	3%	1	1%	83	6%
Projectiles	16	2%	249	81%	3	3%	269	20%
Other or wrong	34	4%	14	5%	33	38%	83	6%
**Total**	**947**	-	**309**	-	**88**	-	**1,358**	**-**

Table 5: Encountered UXO (only identified objects).

Noteworthy is the difference between the type of UXO that different activities have encountered: aerial bombs (mainly by fishermen), projectiles (mainly by dredgers) and mines (relatively often by surveys). An explanation could be the different areas where the activities take place. Another possible explanation is that not all objects are noticed or called in. For example, dredgers may not notice aerial bombs or mines because the grid blocks larger objects. This example also shows another caveat of this study: some usual and relatively cheap safety measures may be effective enough for the risk to have vaporized already, without a dataset to prove this.

[7] We do not include 'other' and 'military' as a result of the limited number of registered instances.
[8] 67 of the 217 mines are ground mines, 134 are contact mines and 16 are 'type unknown'. Approximately 33% of the mines are ground mines and approximately 67% are contact mines.
